# The Role of Mitochondrial DNA Mutations in Mammalian Aging

**DOI:** 10.1371/journal.pgen.0030024

**Published:** 2007-02-23

**Authors:** Gregory C Kujoth, Patrick C Bradshaw, Suraiya Haroon, Tomas A Prolla

**Affiliations:** Baylor College of Medicine, United States of America

## Abstract

Mitochondrial DNA (mtDNA) accumulates both base-substitution mutations and deletions with aging in several tissues in mammals. Here, we examine the evidence supporting a causative role for mtDNA mutations in mammalian aging. We describe and compare human diseases and mouse models associated with mitochondrial genome instability. We also discuss potential mechanisms for the generation of these mutations and the means by which they may mediate their pathological consequences. Strategies for slowing the accumulation and attenuating the effects of mtDNA mutations are discussed.

## Introduction

The mitochondrial theory of aging is based on the premise that reactive oxygen species (ROS), generated throughout the lifespan of an organism, damage mitochondrial macromolecules, including proteins, lipids, and mtDNA. Although most molecular damage is reversible through repair or molecular turnover mechanisms, unrepaired DNA damage may lead to mutations that accumulate as a function of age. The accumulation of mutations ultimately leads to permanent age-related mitochondrial dysfunction, which contributes to the aging phenotype. The mammalian mitochondrial genome is compact (~16 kbp), encoding 13 essential subunits of the respiratory chain and multiple tRNAs and rRNAs. Because cells may have hundreds of mitochondria, and each carries multiple copies of mtDNA, the contribution of mtDNA mutations and deletions to normal aging remains a controversial issue.

## Evidence for a Causal Role of mtDNA Mutations in Aging

Because the most obvious consequence of mtDNA mutations is an impairment of energy metabolism, most studies addressing aging effects have focused on tissues that are postmitotic and display high energetic demands, such as the heart, skeletal muscle, and the brain. Indeed, several studies have unambiguously demonstrated that mtDNA base-substitution mutations accumulate as a result of aging in a variety of tissues and species, including rodents, rhesus monkeys, and humans. In humans, initial studies focused on quantification of individual base-substitution mutations in mtDNA that were shown previously to be pathological in human inherited mitochondrial diseases. For example, the A3243G mtDNA mutation, which results in maternally inherited mitochondrial encephalomyopathy, lactic acidosis, and stroke-like episodes syndrome (MELAS), increases with age in the skeletal muscle of normal humans [1], but only a small fraction of mtDNA molecules in phenotypically normal humans is likely to carry these disease-associated mutations. Thus, it is unlikely that these mutations have deleterious consequences in normal aging. Studies performed in the Attardi laboratory have established that some specific base-substitution mutations can reach high levels in fibroblast cells derived from aged individuals [2] and also in skeletal muscle [3]. The reason why these specific mutations accumulate in mtDNA is unclear, but they are tissue-specific and occur in mtDNA control sites for replication. Interestingly, the same group has found a C150T transition mutation that occurs in most or all mtDNA molecules (i.e., a homoplasmic mutation) is present in leukocytes from approximately 17% of individuals aged 99–106 years old. This mutation is associated with a new replication origin position, suggesting that it may confer a survival advantage in humans [4].

With the development of high-throughput sequencing methods, an unbiased large-scale examination of either selected regions or the entire mtDNA sequence has become feasible. Using a PCR-based amplification and subsequent cloning and sequencing of individual mtDNA fragments, Lin et al. reported that the brains of elderly human subjects had a high aggregate of mtDNA base-substitution mutations, reaching 2 × 10^−4^ mutations/bp [5]. Several studies in rodent and primate tissues are in agreement with this estimate of mtDNA mutational burden, but a study using direct cloning of mtDNA reported much lower levels [6]. This suggests that technical issues remain a problem in determining mtDNA mutation frequencies. Deletions, which can be readily detected by PCR but are not easily quantified, also increase with aging in multiple tissues in rodents [7] and humans [8,9] and can be clonal, as determined by analysis of individual cardiomyocytes from aged humans [10]. In agreement with the hypothesis that mtDNA deletions contribute to mammalian aging, it has been shown that they accumulate exponentially in several tissues, and do so much faster in short-lived mice as compared to long-lived humans [11].

An ongoing debate in the field relates to the issue of causality: are mtDNA mutations merely markers of biological age, or do they lead to a decline in physiological function that contributes to the aging process? Two important age-related phenotypes have helped to address this issue. A common feature of aging in multiple species, including humans, is the age-related loss of muscle mass, termed sarcopenia. Studies using laser capture microdissection to study single muscle fibers in skeletal muscle from sarcopenic rats have shown that mtDNA deletions colocalize with electron transport system abnormalities, fiber atrophy, and splitting [12]. Interestingly, the mutations are largely clonal and absent from phenotypically normal regions within individual muscle fibers [13]. In a similar study of aged (69–82 years old) human muscle biopsies, an association between a deficiency in the mitochondrially encoded cytochrome c oxidase (COX) and clonally expanded base-substitution mutations and deletions was shown [14]. Perhaps the strongest evidence that clonally expanded mtDNA mutations can be causal in both age-related dysfunction and disease comes from recent studies of neurons present in the substantia nigra region of the human brain. These dopamine-rich, pigmented neurons contain very high levels of mtDNA deletions. Deleted mtDNA molecules are clonal in each neuron, and are associated with respiratory chain deficiency [15]. The level of mtDNA deletions increases with normal aging, and is higher in Parkinson's disease [16]. Cytochrome c oxidase–deficient cells have also been shown to increase with age in both hippocampal pyramidal neurons and choroid plexus epithelial cells [17]. Although these studies do not prove causality, they provide strong evidence in support of the hypothesis that mtDNA deletions contribute to aging in mammals.

A significant gap in our knowledge concerns the mechanisms of age-related clonal expansion of mtDNA base-substitution mutations. Using single-cell sequence analysis, Nekhaeva et al. [10] first reported that a high proportion of human buccal epithelial cells and cardiomyocytes carry clonally expanded mtDNA base-substitution mutations. These clonally expanded mtDNA mutations are abundant in cells of aged individuals and result in very different mtDNA mutational spectra in these two cell types. Specifically, epithelial cells display a mutational hotspot in a homopolymeric C_7–8_ tract, whereas almost all cardiomyocyte mutations were observed within a 30-bp sequence in the control region. This sequence was postulated to represent either a binding site for a mitochondrial protein or a secondary structure of functional importance to mitochondria [10]. Because only a small fraction (~5%) of the mtDNA genome was sequenced in this study, it appears very likely that most human cells carry clonally expanded mtDNA base-substitution mutations.

A recently described observation, the accumulation of mtDNA mutations in human crypt stem cells, has also provided insights on the mechanisms of clonal mtDNA mutation accumulation. Taylor et al. described the high incidence of COX-negative cells in intestinal crypts of aged humans [18], and a more recent study strongly suggests that intestinal crypts carrying mtDNA mutations clonally expand by fission [19]. Interestingly, the pattern of distribution of these cells in individual crypts is not random, suggesting that mutations arising in adult stem cells result in the accumulation of such mutations in the tissue. But how do mtDNA mutations become clonal within a cell in the first place? Because the spectrum of expanded mutations is very different between cardiomyocytes and epithelial cells, different mechanisms of expansion, namely random segregation or positive selection, have been proposed for these cell types [10]. Interestingly, modeling of mtDNA replication in human cells suggests that genetic drift and expansion of mutations that occur in early adult life may account for the abundance of specific mtDNA mutations within individual cells [20]. The finding that the clonal expansion of mtDNA base-substitution mutations is a widespread process in human somatic cells may have profound implications for both aging and age-related diseases.

## Human Disorders Associated with Instability of the Mitochondrial Genome

Normal human aging is a gradual, cumulative process that spans decades and most likely involves multiple mechanisms. Information on the specific contribution of mtDNA instability to human aging can be inferred through the analysis of disorders associated with increased mtDNA mutation or deletion frequency. Tissues most affected by disorders associated with inherited mtDNA mutations are the same tissues markedly affected by normal aging; these include the brain, heart, skeletal muscle, kidney and the endocrine system [21]. Disorders associated with increased levels of mtDNA mutations generally fall into two classes: those associated with specific, maternally-inherited mtDNA mutations; and, those associated with mutations in nucleus-encoded genes important for maintaining the fidelity of mtDNA replication and mtDNA stability. Because disorders in the latter category result in random accumulation of many different mtDNA mutations and deletions, they may better represent the potential consequences of age-related mtDNA mutation accumulation in humans.

**Figure 1:**
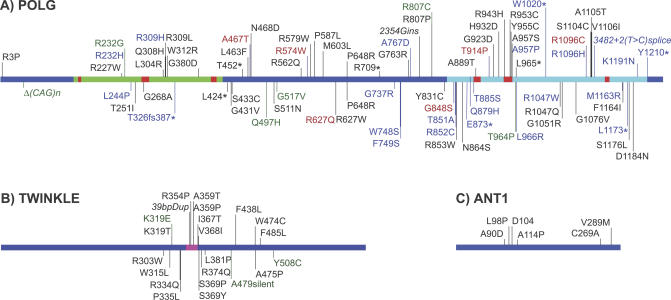
Human Disease-Associated Mutations in Genes Involved in mtDNA Replication and Maintenance Mutations reported in POLG [36,151–162], TWINKLE (gene also known as *PEO1*) [34,38,39,163–167], and ANT1 (gene also known as *SLC25A4*) [35,40] proteins associated with human diseases. Mutations in black are associated with PEO, those in blue are associated with Alpers syndrome, red indicates mutations present in both PEO and Alpers, and green indicates mutations associated with other disorders. Italics indicate changes in DNA sequence. A) POLG. The light green and light blue segments represent the exonuclease and polymerase domains, respectively. Highly conserved motifs within each are shown as red segments. The POLG mutation figure is adapted from the Human DNA Polymerase Gamma Mutation Database maintained by the Mitochondrial Replication Group at the National Institute of Environmental Health Sciences (http://dir-apps.niehs.nih.gov/polg). B) TWINKLE. The pink domain is the primase-helicase linker region, as identified by homology to T7 phage protein [34]. C) ANT1. In addition to the pathogenic mutations shown within the protein, a 3.3-kb deletion upstream of *ANT1* results in derepression of *ANT1* and is associated with facioscapulohumeral muscular dystrophy [168]. Dup, duplication; fs, frameshift mutation; ins, insertion; *, termination codon

Nucleus-encoded DNA polymerase γ (POLG) is the only known DNA polymerase in animal cell mitochondria. It has conserved polymerase and exonuclease domains, the combined action of which results in high-fidelity mtDNA replication with human POLG displaying an average error frequency of ~1 error/500,000 bp in vitro [22,23]. Mutations in the human *POLG* gene are associated with progressive external ophthalmoplegia (PEO), Alpers syndrome, and ataxia (see Figure 1). Disease onset typically occurs after the mid-twenties and can be associated with a variety of symptoms, including ophthalmoplegia, cataracts, progressive muscle weakness, parkinsonism, premature ovarian failure, male infertility, hearing loss (presbycusis), and cardiac dysfunction [24–30]. There are over 80 pathogenic mutations in *POLG* in humans (Figure 1). Most reported mutations are recessive and are commonly found in combination with other mutations in *POLG,* or with mutations in genes encoding other proteins that function in mtDNA replication (such as *TWINKLE* and *ANT1*). At the molecular level, these mutations are often associated with the accumulation of mtDNA deletions in multiple tissues. The few dominant *POLG* mutations reported in PEO occur within the polymerase domain and tend to disrupt the interaction between the polymerase and the incoming nucleotide; this can cause misincorporation of nucleotides and may also lead to large deletions between direct repeats [31,32]. Interestingly, sequencing of mtDNA deletions from patients suggests that replication stalling may be the major mechanism of deletion formation [33].

PEO can also result from mutations in the gene encoding TWINKLE [34], a mitochondrial helicase and putative primase that functions as a hexamer. Mutations in *TWINKLE* are thought to be the cause of 35% of autosomal dominant PEO cases [35]. These mutations seem to enhance dNTPase activity and thus may lower the pool of nucleotides available for mtDNA replication. There are recessive *TWINKLE* mutations that cosegregate with *POLG* mutations, resulting in PEO [36]. Other recessive mutations found in *TWINKLE* and *TWINKY* (a rare splice variant) cause infantile spinocerebellar ataxia [37,38]. Mutations in *ANT1*, an adenine nucleotide translocase involved in ATP/ADP exchange across the mitochondrial inner membrane, as well as mutations in *POLG2*, the POLG accessory subunit, can also lead to PEO [39–41].

In humans, the classical progeroid diseases Hutchinson-Gilford syndrome and Werner's syndrome are associated with defects in nucleus-encoded genes involved in nuclear architecture [42–44] and DNA damage repair [45,46], respectively. The absence of a more general progeroid syndrome in humans carrying mutations that lead to mtDNA instability suggests that mtDNA mutations do not contribute to general aspects of normal human aging. However, the association of genetic disorders of mtDNA instability with cataracts, presbycusis, Parkinson's disease, early menopause, and decreased cardiac and skeletal muscle function suggests that these aging phenotypes are most likely to be influenced by the age-related accumulation of mtDNA base-substitution mutations and deletions.

## Mouse Models of Disease Associated with mtDNA Mutations

A number of transgenic and knock-in mouse models have been developed to test the in vivo effects of increased mtDNA mutation accumulation. Two groups have independently generated knock-in mice expressing an exonuclease-deficient version of the mitochondrial DNA polymerase γ (*Polg^D257A^*) [47,48]. The lack of proofreading activity in *Polg^D257A^* mice results in mitochondrial mutation frequencies that are increased by at least 3- to 11-fold in multiple tissues, with accumulation of mtDNA base-substitution mutations beginning in development. Deletions of mtDNA can also be detected in these mice [48]. The two models have very similar phenotypes resembling aspects of premature aging; these include hair graying and loss, reduced bone density and increased incidence of kyphosis, reduced muscle mass, severe reduction in body fat, early loss of fertility, dilated cardiac hypertrophy, accelerated thymic atrophy, presbycusis, and reduced survival. Anemia and intestinal dysplasia are also seen. A progressive decline in respiratory function of mitochondrially encoded complexes was evident as early as 12 weeks, resulting in decreased oxygen consumption and ATP production [48,49]. No increase in DNA, RNA, protein, or lipid markers of oxidative stress was observed in these mice and antioxidant defense systems were likewise not upregulated [47,49]. Instead, mtDNA mutation accumulation was associated with the activation of apoptosis in multiple tissues as measured by TUNEL and cleaved caspase-3 assays [47].

Additional mitochondrial mutator mouse models have employed tissue-specific *Polg^D181A^* exonuclease-deficient transgenes, expressed primarily in the heart [6] or in the brain [50]. Both base-substitution mutations and mtDNA deletions accumulated in these models. Elevated levels of mtDNA mutations in the heart, beginning after birth, resulted in dilated cardiomyopathy by 4 wk of age, with mutant mice dying of congestive heart failure by ~6 mo [6]. Respiratory function remained comparable to controls [51]. Similarly to the *Polg* knock-in mice, the transgenic hearts did not display increased oxidative damage to proteins (including oxidation-sensitive aconitase enyzme activity) or mtDNA, nor elevated antioxidant defenses [52]. Cytosolic fractions from transgenic hearts contained cytochrome c [51], mitochondrial release of which is a hallmark of apoptosis. Interestingly, although apoptotic (TUNEL-positive and morphologically dying) cells in the transgenic hearts exceeded controls by ~3 wk of age [51], a subsequent protective antiapoptotic response involving upregulation of *Bcl-2, Bcl-xL, Bfl-1, XIAP,* and *Hsp27* transcript or protein levels was noted in nearly all transgenic myocytes [52,53]. This survival response was of functional consequence, in that it could protect the transgenic hearts from further apoptotic stress induced by doxorubicin [53]. This suggests that mtDNA base-substitution mutations and/or deletions in the heart may trigger a retrograde signaling system from the mitochondria to the nucleus. Alternatively, those cells with the highest mutational burden could release cell-extrinsic factors that induce widespread gene expression changes throughout the heart. Cell death seems to be a key element driving the pathology of mtDNA mutations in the heart because cyclosporin A, a cell-death inhibitor that blocks the opening of the mitochondrial permeability transition pore, prevents the cardiomyopathy of the transgenic mice [54].

As discussed earlier, mutations in human *POLG* are associated with chronic PEO, with some patients exhibiting mood disorders [29,30]. Furthermore, mitochondrial dysfunction and altered energy metabolism have been implicated in the etiology of bipolar disorder by magnetic resonance spectroscopy, mtDNA polymorphism association, and detection of mtDNA deletions in bipolar patient brains (for reviews, see [55–57]). In mice with neuronal expression of proofreading-deficient *Polg^D181A^* (under control of the Ca^2+^/calmodulin-activated protein kinase IIα promoter, CaMKIIα), behavioral phenotypes resembling mood disorder were observed, including reduced wheel-running and altered day–night activity patterns [50]. These behaviors were worsened by treatment with amitriptyline hydrochloride, an antidepressant that can induce mania in individuals with bipolar disorder. Although total wheel-running activity decreased, a 5-d pattern of peak activity coinciding with the estrus cycle was observed in female transgenic mice; treatment with lithium, commonly used as a mood stabilizer in the treatment of bipolar disorder, diminished this periodicity. No measurements of respiratory function, apoptosis, or oxidative stress were reported for this model.

In addition to *Polg,* mtDNA replication and maintenance involves the activities of other nuclear genes such as *Tfam* (mitochondrial transcription factor A) [58,59], *Twinkle* (also known as *Peo1* or *C10Orf2*) [60], *Tfb1m* (mitochondrial transcription factor B1, previously called mtTFB) [61], *RNaseH1* [62], and *Ssbp1* (mitochondrial single-strand binding protein; also called *mtSSB*) [63]. Mutations in these genes result in reduction or loss of mtDNA content and mice deficient for some of these genes die during development [58,62]. For example, several mouse models with general [58] or tissue-specific [64–67] deficiencies in *Tfam* have been generated, all based on a *loxP*-flanked *Tfam* allele (*Tfam^loxP^*), and are associated with mtDNA depletion (see Table 1). All of these *Tfam* mouse models exhibit a delay between onset of *cre* expression and the occurrence of respiratory dysfunction, which can be attributed to the time needed to turn over Tfam, mtDNA, and respiratory enzyme subunits.

**Table 1:**
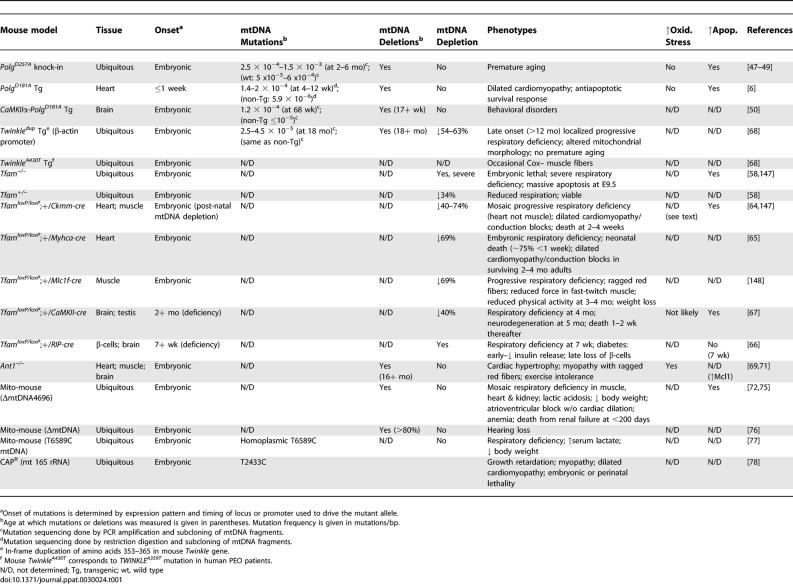
Mouse Models with mtDNA Mutations, Deletions, or Depletion

Pathogenic mutations in *TWINKLE* have been identified in human PEO families [34]. Transgenic mice expressing mutant *Twinkle* isoforms modeled after those mutations seen in human disease display progressive localized mitochondrial respiratory deficiencies, particularly in individual muscle fibers and neuronal subpopulations (transgene expression was noted in heart, muscle, and brain), and mild myopathy at about 1 y of age [68]. These mice acquire multiple mtDNA deletions but do not show increased mtDNA base-substitution mutations. Premature aging does not appear to be a feature of these *Twinkle* transgenic mice, although it is unclear if transgene expression was achieved in most tissues or cell types.

Similarly, mutations in the heart- and muscle-specific isoform of the adenine nucleotide transporter 1 (*ANT1*) gene are present in human PEO families [41]. Disruption of *Ant1* inhibits oxidative phosphorylation by compromising exchange of ADP and ATP across the inner mitochondrial membrane. *Ant1^–/–^* mice display cardiomyopathy and peripheral myopathy with ragged red fibers (a histological marker of mitochondrial proliferation), severe respiratory defects (although electron transport enzyme activities *per se* are intact), elevated serum lactate levels, and exercise intolerance [69]. Increased H_2_O_2_ production in heart, muscle, and brain was observed in *Ant1^–/–^* mice (in addition to high levels in heart and muscle, *Ant1* is expressed at lower levels in the brain and a few other tissues [70]), and was accompanied by varying levels of augmented antioxidant enzymes, depending upon the tissue [71]. Accumulation of mtDNA deletions or rearrangements was observed, with levels in line with the extent of induced antioxidant defenses. In the heart, which showed maximal H_2_O_2_ production (i.e*.*, antimycin A treatment did not further increase ROS levels in the *Ant1^–/–^* tissue) and minimal induction of Sod2 and Gpx1 defense enzymes, mtDNA deletions increased to considerably higher levels than in skeletal muscle, where antioxidant defenses were more robust.

An additional mouse model carrying mtDNA deletions has been generated via a methodology distinct from gene targeting in mouse embryonic stem cells. The so-called “mito-mouse” was generated when synaptosomes (presynaptic terminals isolated after subcellular fractionation) containing mitochondria from aged C57Bl/6J mouse brain were fused to rho° cells devoid of mtDNA. The resulting cytoplasmic hybrid cells (cybrids) were screened by PCR to identify those containing a high proportion of mtDNA deletions. Such cybrid clones were enucleated and fused to donor embryos to create heteroplasmic founder females that could transmit the mtDNA deletion–containing mitochondria through their germline [72]. Germline transmission of mtDNA deletions in humans is rare [73], and partially duplicated mtDNA intermediates were postulated to allow for such transmission in the mice [72]. Mito-mice carry a 4,696 bp deletion in mtDNA that removes six tRNA and seven structural genes (for complexes I, IV, and V) from the mitochondrial genome. F_1_ and F_2_ generations of mice contained varying proportions of the ΔmtDNA4696 deletion and exhibited COX-negative muscle fibers when deletion levels rose to >85%; classic ragged red fibers were not observed, however. Similar mosaic respiratory deficits were noted in heart and kidney. An atrioventricular conduction block was reported, but in the absence of cardiac dilation [74]. Mito-mice were anemic and died from renal failure by 200 days of age. No phenotypes traditionally present in mitochondrial disease or aging were reported. An increase in TUNEL staining was seen in the kidneys of mito-mice, implicating apoptosis as an important mechanism of pathology [75]. A second mito-mouse model with >80% deleted mtDNA exhibited age-related hearing loss with onset between 3 mo and 6 mo [76]. The molecular nature of the mtDNA deletions was not characterized.

Generation of transmitochondrial mice has also been extended to include mtDNA base-substitution mutations. Mito-mice with homoplasmic T6589C-mutated mtDNA, encoding a V421A substitution in the *COI* gene (a COX subunit), show specific loss of COX activity, increased serum lactate, and lower body weight [77]. No further characterization of aging phenotypes is available yet. Transmitochondrial mice bearing T2433C 16S rRNA–mutated mtDNAs (denoted CAP^R^ mice, because the mutation confers resistance to chloramphenicol) displayed growth retardation, myopathy, dilated cardiomyopathy, and embryonic or perinatal lethality [78]. These models of homoplasmic mtDNA base-substitution mutations are more reflective of the inherited mitochondrial disease situation in humans, as opposed to the more random accumulation of mutations and deletions that occurs in normal aging.

Although direct comparisons of mouse models derived through gene targeting, insertional transgenesis, and cybrid approaches is complicated by differences in gene dosage and tissue-specific expression patterns, it is curious to note that multi-system aging-like phenotypes are much more obvious in models bearing increased base-substitution mutations and deletions such as *Polg^D257A^* mice, as opposed to those with only increased deletions (see Table 1). Whether this is biologically meaningful or reflects technical differences in the methodology of mouse generation remains to be determined. Two issues are in particular need of clarification because they could explain the observed differences: First, do the mouse models showing aging phenotypes result in more extensive accumulation of mutations/deletions? And second, do the “ubiquitously expressed” transgenic models truly result in widespread transgenic expression in multiple tissues and cell types?

## Mechanisms of mtDNA Mutation Generation

### Base-substitution mutations caused by polymerase infidelity.

The mitochondrial polymerase γ holoenzyme consists of two separate proteins, POLG and POLG2. POLG, the catalytic subunit, contains the polymerase domain, an editing exonuclease domain, as well as a deoxyribose phosphate lyase activity necessary for DNA repair. POLG2, the accessory subunit, increases the affinity of the complex for DNA, elevating polymerase processivity [79] and repair [80]. The human holoenzyme consists of a heterotrimer of two accessory subunits attached to one catalytic subunit [81]. Detailed kinetics experiments with and without the accessory subunit and exonuclease domain have yielded important insights into the mechanism of polymerase fidelity [23,82,83]. It is important to note that polymerase infidelity has been hypothesized to be the major cause of mutation in human mtDNA and may be responsible for many of the mutational hotspots that appear across individuals [84].

Although in vitro the POLG exonuclease domain plays only a small role in the overall fidelity of the enzyme as compared to the discrimination between incoming dNTPs by the catalytic domain [82], this proofreading activity has been demonstrated to be essential in preventing the accumulation of mutations with age in mice [6,47,48,50] and human cells in culture [85]. Overexpression of the exonuclease-deficient protein in human cells had a dominant negative effect, resulting in the accumulation of mtDNA base-substitution mutations over time. After 3 mo in culture, one mutation was found for every 1,700 bp of mtDNA [85]. These results demonstrate the importance of proper proofreading to prevent mtDNA base-substitution mutations that cause cell and tissue dysfunction with age.

The vast majority of DNA polymorphisms and disease-causing base-substitution mutations that have been detected in human mtDNA are transition mutations [86]. Transition mutations are also the predominant type of mutation in both wild-type and *Polg^D257A^* mice [47,48]. This can be partly explained by the slight infidelity of the POLG enzyme, which allows G:T mismatches to occur as a relatively frequent event [83]. These particular misincorporation events can be exacerbated by dNTP pool imbalances. As shown in rats, dGTP is present at a much higher concentration than dATP in mitochondria from many postmitotic tissues, including heart and skeletal muscle, possibly increasing the frequency of G:T mismatches [87]. In contrast, dTTP is present at the lowest concentration of the four deoxynucleotides in mitochondria from these tissues. These pool imbalances do not differ between young and old animals. At this time, it is unknown what role dNTP pool imbalances play in the generation of the other specific types of mtDNA mutations that occur with age, such as transversion or deletion mutations.

### Oxidative damage to mitochondrial DNA.

mtDNA has been shown to replicate by two distinct mechanisms. In the traditional strand-asynchronous model, replication begins at the heavy (guanine-rich) strand origin and proceeds approximately two-thirds of the way around the mitochondrial genome before initiation of light (cytosine-rich) strand synthesis begins [88]. There is a positive correlation between the rate of accumulation of base-substitution mutations in mammalian mitochondrial genomes and the distance from the origin of light strand replication, relating to the amount of time mtDNA is single stranded during replication [89]. This suggests that mtDNA may be particularly susceptible to oxidative damage when single stranded. Pathogenic mitochondrial base-substitution mutations are found at a disproportionately high level in mitochondrial tRNA genes and it has been hypothesized that this high frequency is due to a stem-loop structure formed when these regions are single stranded during mtDNA replication [90]. However, further evidence is needed to support or refute this suggestion. The spectrum of base-substitution mutations that accumulate in aged individuals differs across tissues [3]. This may be due to variations in the mechanism of replication in different tissues. Specifically, evidence for coupled leading and lagging strand mtDNA synthesis has emerged in recent years [91]. If coupled-strand replication differs from the strand-asynchronous mechanism in its susceptibility to mutation generation, then differential reliance on the two modes of replication among tissue types or under different cellular conditions might contribute to tissue-specific mutation patterns. Alternately, even when replication proceeds primarily via the strand-asynchronous model, utilization of alternate origins of light strand replication may influence mutation specificity by variations in proximity to the heavy strand replication origin and, thus, differences in the time that mtDNA is present in single-stranded form [88].

Mitochondria do not have the enzymes necessary for nucleotide excision repair of DNA. They do, however, possess base excision repair enzymes that are capable of repairing oxidatively damaged bases in mtDNA, and many of these repair enzymes are alternatively spliced variants of nucleus-targeted proteins [92]. Mitochondrial base excision repair activity declines in the aging mouse brain [93]; if applicable to tissues in general, this may contribute to the accumulation of mtDNA mutations with age. Base-substitution mutations may occur as a result of POLG replicating across these lesions. In mitochondria, 8-oxoguanine is the most abundant oxidative lesion and can cause transversion mutations if unrepaired [94]. Mitochondria contain an 8-oxoguanine DNA glycosylase (OGG1) that repairs the vast majority of this damage. Mice lacking Ogg1 had 20-fold higher levels of 8-oxoguanine in mtDNA isolated from liver [95] but this did not lead to respiratory defects [96]. Mitochondria also possess an 8-oxoGTPase (MTH1) to prevent oxidized dGTP from being incorporated into DNA [97]. mtDNA in brains from mice lacking Mth1 accumulate more 8-oxoguanine than controls [98,99]. No accelerated aging phenotypes were observed in either *Ogg1^–/–^* [95,100] or *Mth*1^–/–^ mice, although both are slightly more prone to certain types of tumors [101,102]. Unexpectedly, *Ogg1^–/–^Mth*1^–/–^ mice have a decreased incidence of tumorigenesis [101]. Both OGG1 and MTH1 also function outside mitochondria to protect nuclear DNA, so it is unclear if the phenotypes of these mice are related to mtDNA or nuclear DNA mutations.

### Mitochondrial DNA deletions.

mtDNA deletions may play a contributing role in age-related tissue dysfunction in human postmitotic tissues. Deleted mtDNA molecules can accumulate, reaching up to 60% of the total mtDNA and cause oxidative phosphorylation defects and COX-negative staining in specific cells of aged postmitotic tissues [14,15]. The mechanism of deletion formation is unknown. However, many deletions are thought to involve base pairing by direct repeat sequences [103] and this occurs more frequently during oxidative stress, perhaps due to polymerase stalling, slipping, and mispairing during replication (Table 2). Topoisomerase II cleavage and other DNA double strand breaks have also been proposed as possible mechanisms of deletion formation [103,104]. A study analyzing deletions in human mtDNA suggests that most deletion formation may be linked to two 13-bp repeats in mtDNA [105].

**Table 2:**
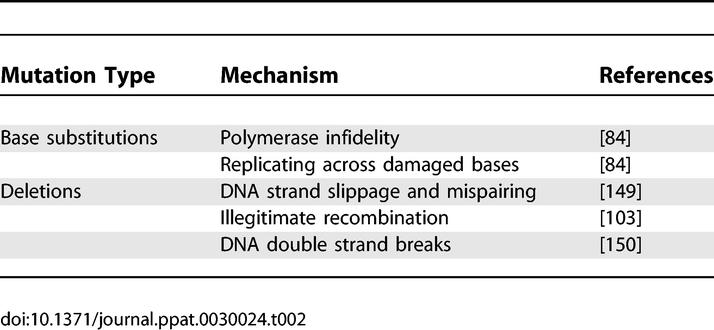
Mechanisms for mtDNA Mutation

## Mechanisms of Pathology Induced by mtDNA Mutations

Data from mitochondrial mutator mouse models support the hypothesis that mtDNA mutations can promote tissue dysfunction through the loss of critical irreplaceable cells due to activation of apoptosis. In support of this hypothesis, human cells bearing mutations causing Leber's hereditary optic neuropathy, an inherited mtDNA disease, are sensitized to Fas-induced apoptosis [106]. Is apoptosis *required* for development of mtDNA-induced phenotypes, and how might mtDNA mutations trigger the apoptotic process? Loss of respiratory function is associated with activation of apoptosis (e.g., see mouse models of mtDNA depletion in Table 1), and mitochondrial bioenergetics are compromised in mitochondrial mutator mice [48,49]. Release of apoptotic factors, such as cytochrome c, Smac/diablo, apoptosis-inducing factor (AIF), Omi/Htra2, and endonuclease G from the mitochondrial intermembrane space can occur through two mechanisms [107,108]. In the first, channels in the outer mitochondrial membrane can open in a process regulated by Bcl-2 family members without the involvement of inner mitochondrial membrane components. In the second, opening of a permeablility transition (PT) pore, involving components of the outer mitochondrial membrane (VDAC, Bax, and Bcl-2), inner mitochondrial membrane (ANT), and matrix (Cyp D) results in osmotic mitochondrial swelling, outer mitochondrial membrane rupture, and release of apoptogenic factors. The observation that cyclosporin A–mediated inhibition of PT pore opening was successful in preventing cardiomyopathy in the heart-specific mitochondrial mutator model [54] implicates a central role for the PT pore generally, and cyclophilin D (Cyp D) in particular, in mtDNA mutation-mediated cell-death signaling in the heart, because Cyp D is the main mitochondrial binding target of cyclosporin A [109]. However, mitochondria from the *Polg^D181A^* transgenic hearts are purportedly more resistant to calcium-induced PT pore opening than those from control hearts [110], an effect attributed to the protective actions of induced Bcl-2 in the pro-survival response. Thus, other functions of Cyp D aside from its role in PT pore opening (such as its chaperone activity) may be important. Mouse models deficient for many of the genes involved with apoptotic regulation (e.g., *Bax, Bak,* and *Cyp D*) are available. Examining the effects of these apoptotic modulators on the aging phenotypes of mitochondrial mutator mice should help to establish whether apoptosis is required for the downstream effects of mitochondrial mutations.

Recently, Zassenhaus and colleagues proposed an intriguing mechanism whereby mtDNA mutations would generate a pool of misfolded mitochondrial proteins, some small proportion of which might have the conformation necessary to bind to Bax or Bak and thereby activate apoptosis or perhaps bind to Cyp D and inhibit its chaperone function [110]. This hypothesis could explain how heteroplasmic mtDNA mutations could elicit a cell-death response in the presence of many wild-type copies of mtDNA.

A long-standing tenet of the mitochondrial free radical theory of aging is the expectation of increased ROS production in mitochondria compromised by respiration-inactivating mtDNA mutations (i.e., “the vicious cycle”). However, we [47] and others [49,52] have clearly demonstrated that mitochondrial mutator mice do not have increased levels of oxidative stress. Mitochondria treated with specific chemical electron transport chain (ETC) inhibitors can indeed produce increased ROS levels [111]. Similarly, mouse models such as the *Ant1^–/–^* mice also exhibit elevated levels of ROS production [71]. However, inhibition of ETC function in *Ant1^–/–^* mice or by chemical inhibitors may generate ROS because all mitochondria show the same defect (e.g., lack of available ADP or blockage of electron flow at a specific point in the ETC). Upstream complexes can still function, resulting in electron stalling and transfer to O_2_ to generate the superoxide anion. By contrast, in the mitochondrial mutator mice, a variety of mutations is present and multiple upstream complexes could be nonfunctional or be lacking subunits if mitochondrial rRNA or tRNA mutations are numerous. Thus, electron flow through all the complexes (except nucleus-encoded complex II) may be impaired and reduced intermediates may not be accumulating. In the case where mtDNA mutation levels are much lower, the presence of many wild-type copies of mtDNA will mask the effects of specific respiratory mutations.

If mtDNA mutations do not lead to increased ROS damage in mitochondrial mutator mice, how does this finding fit into the field of oxidative stress and aging? Certainly, oxidative stress could be playing a role in the generation of mtDNA mutations in wild-type animals. The rate of mitochondrial ROS production, extent of mtDNA (but not nuclear DNA) oxidative damage, and degree of membrane fatty acid unsaturation (a determinant of vulnerability to lipid peroxidation) are all inversely correlated with longevity across species [112–115]. Most of these parameters are reversed by caloric restriction (CR) [116]. Mice expressing mitochondrion-targeted catalase show reduced total DNA oxidative damage (in skeletal muscle), fewer mtDNA deletions, and extended mean and maximal lifespan by 17%–21% [117], suggesting that mitochondrial accumulation of oxidative damage can limit rodent lifespan. However, mice with reduced levels of the mitochondrial MnSOD enzyme (*Sod2^+/–^*) do not appear to age any faster than their wild-type counterparts, despite harboring increased levels of oxidative damage to both nuclear and mtDNA [118]. Similarly, mice deficient for Ogg1 or Mth1 do not exhibit accelerated aging features [95,100,102]. Thus, increased mitochondrial oxidative damage is not sufficient for accelerated aging. It is unclear, however, whether the increased oxidative damage to mtDNA observed in the *Sod2* ^+/−^ mouse model actually leads to increased base substitutions or deletions, and, if so, to what extent the mutation levels compare to those of the Polg mutator mice or natural aging.

The mitochondrial mutator mice suggest that activation of apoptotic pathways is important for the induction of an aging phenotype. Indeed, we speculate that activation of apoptosis may be a common underlying mechanism in many accelerated aging models. For example, mice that are both deficient in Werner's *(Wrn)* helicase and possess shortened telomeres display a phenotype strikingly similar to *Polg^D257A^* mice and exhibit elevated levels of apoptosis [119]. Livers from old, but not young or middle-aged, *Sod2^+/–^* mice have 3-fold more TUNEL-positive cells [120]. Therefore, the long delay before activation of apoptosis in the *Sod2^+/–^* mice might account for the failure to see early aging phenotypes in these animals.

The phenotypes of multiple mouse models of mtDNA base-substitution or deletion mutation accumulation that have been generated in recent years have lent support to the notion that mtDNA mutations can play a causative role in aging-related degenerative processes. This interpretation is not without controversy, however, with opponents arguing that the levels of mtDNA mutations present in the mitochondrial mutator mice are catastrophically high and exceed those associated with human aging [121–123]. The manifestation of clinical phenotypes of classic mitochondrial diseases are dependent upon mtDNA mutations or deletions rising above a critical threshold, so one might also expect that aging phenotypes require accumulation of mutations exceeding a threshold. High mtDNA mutational loads accompanied by severe respiratory deficiency have been observed in muscle fibers, intestinal crypts, and substantia nigra neurons [2,3,5,15,16,18,19]. Ultimately, testing the effect of reduced mtDNA mutation accumulation on lifespan and aging phenotypes will provide the strongest support of a causal relationship between mtDNA mutations and aging.

**Figure 2:**
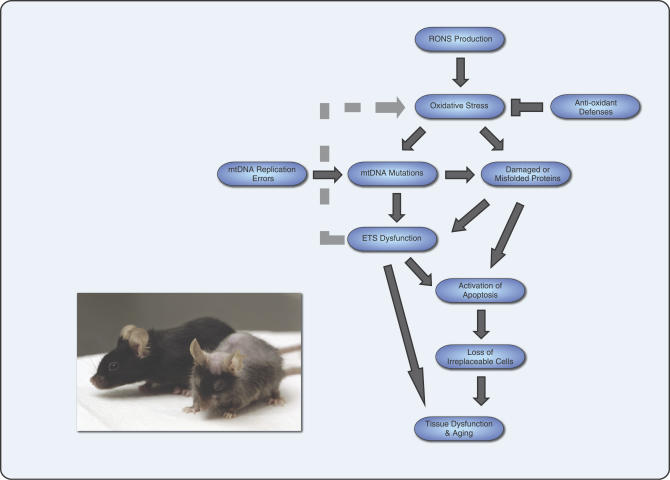
Multifactorial Events in Mammalian Aging Oxidative stress is generated when reactive oxygen and nitrogen species (RONS) production exceeds protection from antioxidant defenses, and can lead to damaged proteins and mtDNA mutations. Replication errors are an additional source of mtDNA mutations. mtDNA mutations can result in reduction or loss of respiratory complex function and a pool of aberrant mitochondrial proteins. In certain situations (e.g., homoplasmic inherited mtDNA base-substitution mutations), specific mtDNA mutations could lead to increased oxidative stress, but this is not a feature of mice bearing random accumulations of mtDNA mutations. Activation of apoptosis could occur through mechanisms that sense energetic deficits or by signaling via rare misfolded proteins that might be capable of interacting with apoptotic regulators such as Bax or Bak. Prolonged activation of apoptotic cell death would gradually deplete tissues of both differentiated and possibily regenerative stem cells, leading to eventual tissue dysfunction and aging-related phenotypes. Additionally, chronic energetic deficiency in itself may contribute to altered tissue functioning with age. Inset: *Polg^D257A^* mitochondrial mutator mouse (right) showing hair graying, alopecia, and kyphosis compared to a healthy age-matched control mouse. ETS, electron transport system; RONS, reactive oxygen and nitrogen species

It is important to note that aging is a complex process that is likely to have multifactorial causes (Figure 2). Mitchondrial DNA mutations can arise directly from errors during DNA replication. Oxidative stress may also generate mtDNA mutations as well as damaged proteins that might be able to directly signal apoptosis through a misfolded protein response. Respiratory deficiency could contribute to apoptotic signaling or be directly responsible for some aspects of tissue dysfunction. The importance of cell loss versus metabolic dysfunction to aging phenotypes might vary depending upon the tissue type.

## Interventions to Retard mtDNA Mutations and Its Consequences

Because mtDNA mutations cause dysfunction in cells, it is of interest to determine if preventing these mutations could delay the onset or decrease the severity of aging phenotypes. Several strategies have the potential to retard age-related accumulation of mtDNA mutations (Table 3).

**Table 3:**
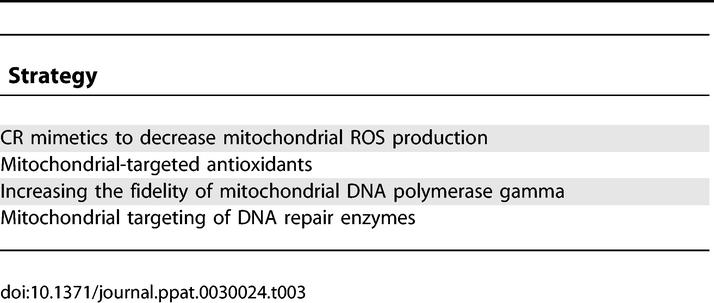
Strategies to Slow the Age-Related Increase in mtDNA Mutations

### 

#### Decreasing the generation of ROS by caloric restriction.

The only known intervention that has consistently delayed aging in multiple species is CR [124]. Decreased levels of mtDNA deletions are detected in calorically restricted animals [125]. The mechanism of protection by CR may involve decreased production of ROS from Complex I of the mitochondrial ETC [126]. Although this involves altering the degree of reduction of Complex I, the exact mechanism as to how this occurs is unknown. One hypothesis is that decreased levels of methionine ingested during CR lead to a higher reduced/oxidized cellular glutathione ratio, which decreases Complex I free radical generation [127]. Another hypothesis is that mitochondria from calorically restricted animals undergo increased biogenesis, and are more efficient than normal at generating equal amounts of ATP with a lower membrane potential, oxygen consumption, and free radical production [128].

#### Decreasing oxidative damage with antioxidants.

The use of nutritional and genetically encoded antioxidants can prevent mtDNA mutations. Expression of mitochondrion-targeted catalase, which decreases hydrogen peroxide levels, prevented mtDNA deletions and extended the lifespan of mice [117]. Nutritional antioxidants may function, in part, by maintaining mitochondrial glutathione in the reduced state, which can prevent the increase in free radical generation from the ETC that occurs with age and damages mtDNA [129]. Examples of dietary antioxidants that decrease the accumulation of potentially mutagenic 8-oxoguanosine levels in mtDNA include carnitine [130], alpha tocopherol (Vitamin E) [131], Vitamins C and E in combination [129], thiazolidine carboxylate [129], and Ginko biloba extract [132]. Oxidative stress also leads to deletions in mtDNA that can be prevented by dietary antioxidants. Beta-carotene and creatine protected against mtDNA deletions in skin human fibroblasts exposed to UVA radiation [133,134], while coenzyme Q lowered the level of mtDNA deletions in a mouse model of oxidative stress [135]. Interestingly, dihydrolipoic acid partially rescued the phenotype of yeast cells expressing a POLG variant carrying a mutation found in PEO patients [136]. However, none of these antioxidant compounds has yet consistently been shown to be beneficial in the treatment of human mitochondrial disease [137], and likewise none has been shown to retard mammalian aging.

#### Increasing mitochondrial DNA repair.

There is evidence in cell culture that expression and mitochondrial targeting of base excision repair enzymes protects against oxidant-induced cell death. Proteins found to be protective include human 8-oxoguanine DNA glycosylase/apurinic lyase (OGG1) [138] and E. coli endonucleases III and VIII [139]. The MTH1 protein, a free 8-oxo-GTPase present in both the nucleus and mitochondria, also protects cells from oxidative stress [140]. Extensive studies examining the roles of these proteins in aging have yet to be performed and may yield insight into possible connections between mtDNA damage and aging.

It has been reported that liver mitochondria contain a DNA mismatch repair activity [141], a pathway that corrects DNA polymerase errors and inhibits other kinds of genome instability. Consistent with this observation, the DNA mismatch repair enzyme Mlh1 has been localized to mouse mitochondria [142,143]. There have also been mixed reports on the localization of the mismatch repair protein Msh2 in mitochondria [141,143]. Bioinformatic analysis implicates Msh5 as a candidate mtDNA repair enzyme [142]. Whether or not these enzymes actually constitute a functional mitochondrial mismatch repair system awaits further verification. Yeast and plant mitochondria utilize the MSH1 protein in mitochondrial mismatch repair, whereas no MSH1 homolog is present in mammalian mitochondria [144]. Increased expression of one or more of these proteins in mitochondria might have the potential to delay the accumulation of mtDNA mutations with age.

## Conclusion

The hypothesis that aging is due in part to mtDNA damage and associated mutations [145,146] was based on the observations that mtDNA is located in the organelle that generates most cellular ROS, that mtDNA is relatively unprotected from ROS damage due to a lack of histones, and also that mtDNA repair may be limited. Although provocative, this hypothesis is only viable as a major aging mechanism if three conditions are met for any given tissue: 1) mutations must accumulate with age; 2) due to the high copy number of mtDNA, most mutations should reach near or complete homoplasmy; and 3) such mutations must be of functional consequence. The first two conditions have clearly been satisfied for several cell types examined in humans. Future developments in the field are likely to focus on identifying the functional consequences of specific mtDNA mutations found in aged human tissues, mechanisms of clonal expansion, and the dissection of pathways that mediate the deleterious effects of mtDNA base-substitution mutations and deletions using animal models. These studies should help uncover the relevance of mtDNA mutations to animal aging, and allow the rational design of therapeutic interventions. 

## Accession Numbers

The National Center for Biotechnology Information Entrez Gene (http://www.ncbi.nlm.nih.gov/entrez/query.fcgi?CMD=search&DB=gene) accession numbers for the genes and gene products discussed in this paper are *POLG* (5428); TWINKLE, or *PEO1* (56652); and *ANT1,* or *SLC25A4* (291).

## Abbreviations

CR: caloric restriction
ETC: electron transport chain
mtDNA: mitochondrial DNA
PEO: progressive external ophthalmoplegia
PT: permeablility transition
ROS: reactive oxygen species
